# Impact of mass drug administration with Ivermectin, Diethylcarbamazine, and Albendazole in elimination of lymphatic filariasis in five districts of Nepal

**DOI:** 10.1371/journal.pgph.0004809

**Published:** 2026-04-24

**Authors:** Ram Kumar Mahato, Gokarna Dahal, Yadu Chandra Ghimire, Rudra Prasad Marasini, David T. S. Hayman, Kaliannagounder Krishnamoorthy, Sunil Raj Sharma, Dipak Sah, Ram Balak Ray, Radha Subedi, Keshav Raj Pandit, Sudip Raj Khatiwada, Achut Babu Ojha, Saroj Mahaseth, Deepak Bahadur Mahata, Molly Brady, Clara R. Burgert-Brucker, Briana Stone, Ashna Parajuli, Satya Raj Paudel, Bhim Prakash Devkota, Chandra Bhal Jha, Krishna Prasad Paudel, Bhim Prasad Sapkota, Bijay Bajracharya

**Affiliations:** 1 Epidemiology and Disease Control Division, Teku, Kathmandu, Nepal; 2 Molecular Epidemiology and Public Health Laboratory, Infectious Disease Research Centre, Hopkirk Research Institute, Massey University, Palmerston North, New Zealand; 3 ICMR-Vector Control Research Centre, Puducherry, India; 4 Vector Borne Disease Research and Training Center, Hetauda, Makwanpur, Nepal; 5 RTI International, Act to End NTDs | East, Kathmandu, Nepal; 6 RTI International, Act to End NTDs | East, Research Triangle Park, North Carolina, United States of America; 7 FAIRMED Foundation, Kathmandu, Nepal; 8 Department of Health Services, Teku, Kathmandu, Nepal; 9 Ministry of Health and Population, Ramshahpath, Kathmandu, Nepal; 10 Center For Health and Disease Studies, Nepal; CSIR-Indian Institute of Chemcial Technology, INDIA

## Abstract

Nepal aims to eliminate lymphatic filariasis (LF) by 2030. Mass drug administration (MDA) has ceased in 53 of the 64 endemic districts. Following failure to pass the pre-Transmission Assessment Survey of antigenemia prevalence, five districts completed two rounds of MDA using a three-drug regimen (Ivermectin, Diethylcarbamazine, and Albendazole; IDA) and achieved over 65% coverage of the total population in 2023 and 2024. An Epidemiological Monitoring Survey (EMS) was conducted to evaluate IDA’s impact. A cross-sectional EMS was conducted 9 months post-MDA in 11 evaluation units (EUs) across five districts, using two sites per EU (n = 22). A total of 6,829 individuals aged ≥20 years were sampled via multi-stage methods, with ≥300 blood samples per site. Data on demographics and MDA participation were collected. LF antigen testing was followed by night blood microfilariae testing in antigen-positive samples. Analysis included non-parametric tests, logistic and mixed-effects models accounting for site-level clustering, and penalized regression (lasso and ridge) to assess predictor importance and manage multicollinearity. Nine of 11 EUs passed EMS. Two EUs in Kapilvastu failed due to ≥1% microfilariae prevalence in at least one site. Microfilariae prevalence was negatively correlated with site MDA coverage (p-value 0.04), but not antigen prevalence (p-value 0.8). Overall, 4.63% of participants were antigen-positive and 0.34% were microfilariae-positive (ratio 14:1). Being female (OR 0.12; 95% CI: 0.04–0.36) and participation in latest MDA round (OR 0.34; 95% CI: 0.15–0.77) were associated with lower microfilariae prevalence. Suboptimal impact of MDA was observed in two EUs based on microfilariae prevalence and may reflect insufficient treatment compliance. Two additional rounds of MDA with directly observed treatment are recommended to improve adherence. Female sex and participation in the most recent MDA round were associated with reduced odds of microfilaremia. Targeted strategies focusing on men and other identified risk groups may enhance program effectiveness.

## Background

Lymphatic Filariasis (LF), a mosquito borne filarial parasitic infection, causes disease conditions such as lymphedema and hydrocele. This disease is recognized globally as a neglected tropical disease (NTD) and remains a major public health problem in many tropical and subtropical countries and is the second leading infectious cause of disability worldwide [1]. LF is targeted for elimination as a public health problem by 2030 using two key strategies: mass drug administration (MDA) with anti-filarial drugs to interrupt transmission and morbidity management and disability prevention (MMDP) to alleviate sufferings among those affected [2].

In 2024, 35 countries were considered to require MDA. In contrast, 37 countries no longer required MDA, including 21 that have met the criteria for elimination as a public health problem [3].

The national LF Elimination Program of Nepal has realigned the initial target of achieving LF elimination by 2020 [4,5] with the WHO’s NTDs Roadmap to meet the elimination target by 2030 [4,5]. Out of 77 districts in Nepal, 64 are endemic for LF with approximately 27.7 million people (95.1% of the total population) at risk of infection at the beginning of the program [4,6].

*Wuchereria bancrofti* transmitted by *Culex quinquefasciatus* is the only infection reported in Nepal [7]. The Epidemiology and Disease Control Division (EDCD) under the Ministry of Health and Population (MOHP) has implemented the LF elimination program since 2003, initially using a two-drug regimen (diethylcarbamazine and albendazole, DA) [8]. As a first step to determine how MDA has affected LF prevalence, WHO recommends sentinel and spot-check site surveys in an evaluation unit (EU); if prevalence is lower than 2% for LF antigen or 1% microfilariae in each site, the EU can progress to implementing a Transmission Assessment Survey (TAS; TAS-1). TASs are population-based cluster antigen surveys of 6–7-year-olds to determine if prevalence is low enough to stop MDA; a TAS should then be repeated two (e.g., TAS-2) and four (e.g., TAS-3) years after stopping MDA [9]. By the end of 2023, MDA was stopped in 53 Nepali districts following successful demonstration of TAS-1. Of these, 47 districts have passed TAS-2 and 28 districts passed TAS-3. In 2022, a triple drug-regimen (ivermectin, diethylcarbamazine and albendazole; IDA) was introduced in five districts (Morang, Kapilvastu, Dang, Banke and Kailali) [10] that repeatedly failed to meet the ‘Pre-TAS’ standards (see below). This regimen was scaled up to 11 districts in 2023 as 10 districts failed Pre-TAS/TAS requirements, and one a newly classified endemic district [10,11].

As per the WHO’s guideline on the monitoring and evaluation of triple drug regimen, an Epidemiological Monitoring Survey (EMS-previously, known as Pre-TAS) should be conducted nine months after the last round of MDA following two effective (>65% epidemiological coverage at district level) rounds of MDA [12] in every EU with a population of not more than 0.5 million. The new survey requires testing individuals for filarial antigen in selected sites and detecting microfilariae (Mf) through night blood smears of all antigen-positive individuals, which differs from the previous practice of surveying only for antigen. EUs that pass the EMS become eligible for the IDA Impact Survey (IIS-equivalent to TAS). Following the successful IIS, two additional IIS (similar to TAS-2 and TAS-3) should be conducted at two-year intervals to ensure that infection levels sustain below the thresholds at least for 4 years [12]. In 2023, we conducted EMSs in 11 EUs following two rounds of IDA in Morang, Kapilvastu, Dang, Banke, Kailali districts and the results are analyzed for their eligibility to MDA stop survey or IIS, having recorded Mf prevalence below the threshold of <1% and to identify key predictors of this outcome.

## Methods

The cross-sectional EMS was conducted using a multi-stage sampling design to determine whether two rounds of IDA MDA could reduce LF infection prevalence to below the transmission threshold, defined as <1% microfilariae prevalence, in sites within the EUs of 5 districts: Morang, Kapilbastu, Dang, Banke, and Kailali. Secondary objectives included evaluating MDA coverage, identifying predictors of LF infection, and comparing the ratio of LF antigen prevalence to microfilariae prevalence. The survey was carried out after 9 months of completion of MDA campaign and between 1^st^ January-30^th^ January 2024.

Stage 1: Five districts were selected where two rounds of IDA MDA had been completed with an effective epidemiological coverage of ≥65% at district level (MDA coverage for each district was determined from final MDA reports from EDCD). The coverage for each district was supported by data from surveys conducted with the WHO-recommended Supervisor’s Coverage Tool (SCT) [13] and Spot Compliance Survey [14], both of which provide proxy measures for evaluating MDA coverage.
Stage 2: The selected districts were divided into EUs, each with a population of not more than 500,000, as per WHO provisional guidelines [6,12]. The EUs were defined based on risk factors, including low MDA coverage in the previous years, high number of resistant populations (i.e., populations that are reluctant to be medicated), high numbers of reported clinical cases, or proximity to low coverage areas. EDCD, in close coordination with partners and district program managers, established two EUs in Kapilbastu, Dang, Banke, and Kailali and 3 EUs in Morang [6].
Stage 3: EUs were the survey area and for sampling, two sites were selected – usually a sentinel site and a spot-check site. If a sentinel site did not exist or the sentinel site had < 2% antigen prevalence in previous surveys, an extra spot-check site was chosen instead. If a spot-check site had an antigen level >2% in a previous survey it was included in this survey; otherwise, a new site was chosen. Ward, the lowest administrative unit in Nepal, served as the site. The sites were selected based on factors such as having high number of clinical cases, vector abundance, and poor epidemiological coverage of IDA MDA, with the assumption that if the most challenging sites had infection prevalence below the transmission threshold, the rest of the EU would also maintain a similar level. The sites selected in the 11 evaluation units and 5 districts are shown in S1 Table.
Stage 4: A total of approximately 300 samples per site, or 600 samples per EU, were required, amounting to around 6,600 samples across 11 EUs. This sample size was calculated to detect a critical threshold of <2% with a 5% chance of Type I error and ~75% power when the true prevalence is 1%, accounting for a design effect of 1.5 for populations under 2,800 or 2.0 for populations over 2,800. In total, 6,829 samples were collected to assess the impact of the LF IDA MDA.

The required number of households to achieve a sample size of 300 adults (population above ≥20 years) per site was calculated, accounting for an expected 25% non-response rate, and an average of 2.65 adults per household [6]. This resulted in the selection of approximately 151 households per site, rounded to the nearest multiple of 50 for determining the segment size. During a ward-level meeting before the field survey, each site was divided into equal segments containing approximately 150 households and one segment was randomly selected from each site. All households within the selected segments were visited, and all available adults aged ≥ 20 years were asked to participate in the survey from a team of enumerators with the help of local health workers and female community health volunteers. Each eligible adult visited a temporary laboratory established nearby and were enrolled and interviewed about participation in the last MDA (did the person participate in the MDA and did they take the IDA) and whether they had ever been treated and tested for LF antigen using a rapid antigen test, Filariasis Test Strip (FTS). Night blood samples were collected for Mf from the antigen positives either on the same day, or the next day in case of absentees as described below. Summary and detailed information on the EUs is in S1-S4 Tables.

### Diagnostic tests

The EMS employed FTS for the detection of circulating filarial antigen (CFA), followed by microfilariae testing for those who tested antigen-positive, to assess any potential transmission signals.

### FTS testing

The FTS was used as the primary diagnostic tool to detect LF antigenemia among the survey population. Antigenaemia surveys were done between 8.00 AM and 4.00 PM. Day blood samples were collected using a 75-μL blood collection pipette after pricking the left ring finger. The blood sample was then transferred to the sample application pad with the pipette, and the result was assessed exactly at 10 minutes. Each FTS was labeled with a unique ID so that tests could be linked with demographic information and other variables collected. FTS Test scores were recorded as negative if no test line was visible (but with a positive control line); and as positive if the test line was present (along with the positive control line). Tests with no control line or other issues such as no migration of blood were considered invalid results [15]. Initially, we found only two antigen tests with invalid results and they were repeated. Both these tests were valid and negative, and the test results were included in the number tested for antigenemia. Invalid results were reported to WHO as per survey protocol. All individuals who tested positive with FTS were further tested for microfilariae through night blood slide collection.

### Microfilariae testing

Microfilariae testing was conducted using microscopy to detect microfilariae among all antigen positive individuals [16]. The microfilariae testing was done either on the same night or the following night after the FTS results were confirmed. Trained lab staff were responsible for preparing one quality blood smear per person (slides), storing, and transporting them to laboratory facilities. The same staff member dried it for 24 hours, stained it properly using Giemsa solution, and read the slides under the microscope for the presence of microfilaria.

#### Sample collection.

Night blood slides were collected between 10:00 PM and 2:00 AM to cover the peak appearance of microfilariae in the peripheral blood [17]. After pricking the finger on the side of the finger pad, 60-μL of blood was collected into a calibrated capillary tube and applied in three parallel lines (approximately 20 µL each) along the length of the microscope slide.

#### Slide preparation and staining.

The slides were air-dried overnight and then placed in distilled water for 5 minutes to dehemoglobinize the blood following standard procedures [9]. After air drying for 1 hour, the slides were fixed in methanol for 5 minutes and stained with a 1:50 dilution of Giemsa stock for 50 minutes. The slides were air-dried again before being read under a microscope using 10X and 40X lenses to detect microfilaria.

#### Quality control.

All microfilariae-positive slides and negative slides were re-read by a second reader. The second reader was from the agency responsible for surveys within the MOHP having ‘Master Training of Trainers’ for LF microscopy and intensive experience of microfilariae microscopy to ensure accuracy.

### Data analysis

Prevalence of those antigen-positive and those microfilariae-positive were calculated for each site. For antigen, number of antigen-positive participants was divided by the number of antigen-positive and antigen-negative participants tested in the site. For microfilariae, the number of microfilariae-positive participants was divided by the sum of the microfilariae-positive participants plus microfilariae-negative participants plus antigen-negative participants (who were assumed to be microfilariae negative).

Odds ratios (ORs) and 95% confidence intervals (CIs) were estimated using univariate logistic regression models for antigenemia (CFA positivity) and microfilaremia. The reference categories were: age 20–29 years for age group comparisons, male for sex, and non-participation in the most recent MDA round for treatment participation. An OR <1 indicates lower odds of infection relative to the reference category.

Differences in age distributions between antigen and microfilariae positive and test negative individuals were assessed using t-tests with Welch’s correction or Wilcoxon rank-sum tests where appropriate using R (v4.3.2). A one-sample non-parametric test using IBM SPPS statistics 25 was employed to calculate 95% CI for proportions of samples positive for antigen and microfilariae. The samples were grouped by age class (20–29, 30–39, 40–49 and 50 and above) and analyzed antigen and microfilariae positives. Percentages were calculated to compare both 1) the positivity rates of LF antigenemia and microfilaremia out of the total number of people sampled, and 2) the percentage of microfilariae test positive out of those antigen positives. Correlation analyses, Chi-square tests (χ^2^) and odds ratios (OR) were used to assess the associated factors with MDA coverage.

Multivariate logistic regression was used to assess associations with microfilaremia, focusing on sex, age, and participation in the most recent MDA round. Because participants were sampled within districts (and sites), observations are not independent. We therefore fitted generalized linear mixed models (GLMMs) with a random intercept for district using the glmer function in the lme4 package (Laplace approximation). For microfilaremia, the GLMM included sex and age as fixed effects and district as a random effect.

To explore potential confounding and effect modification, we initially considered multivariable models including sex, age (continuous), district, and recent MDA participation, including interaction terms. However, microfilaremia was rare and some districts had zero or near-zero positive cases, leading to sparse-data instability and separation in conventional logistic models. Therefore, in addition to the GLMM described above, we fitted penalised logistic regression models (ridge and lasso) using the *glmnet* R package [18] with sex, age, recent MDA participation, and district as a fixed-effect factor. These penalised models were used as shrinkage-based sensitivity analyses to stabilise estimates under sparsity; they do not include random effects and therefore do not explicitly model within-district correlation. The data used in these models is plotted by the predictor variables in S1 Fig and S2 Fig.

### Ethics approval, consent to participate, and consent for publication

This survey was carried out by Epidemiology and Disease Control Division (EDCD) and Vector Borne Disease and Research Center (VBDRTC); both institutions are the major implementing bodies for management of vector borne diseases under Nepal’s Ministry of Health and Population (MoHP). The activities conducted by MoHP as a part of the set strategic goals for the regular monitoring and programmatic progress in National Lymphatic Filariasis Elimination Program (NLFE) have been exempted by Nepal Health Research Council (NHRC) from the ethical review process (**Ref. no 1530 NHRC, December 10, 2020**). Nevertheless, written informed consent was obtained from all the participants prior to interviews and blood sample collection.

## Results

**Fig 1:**
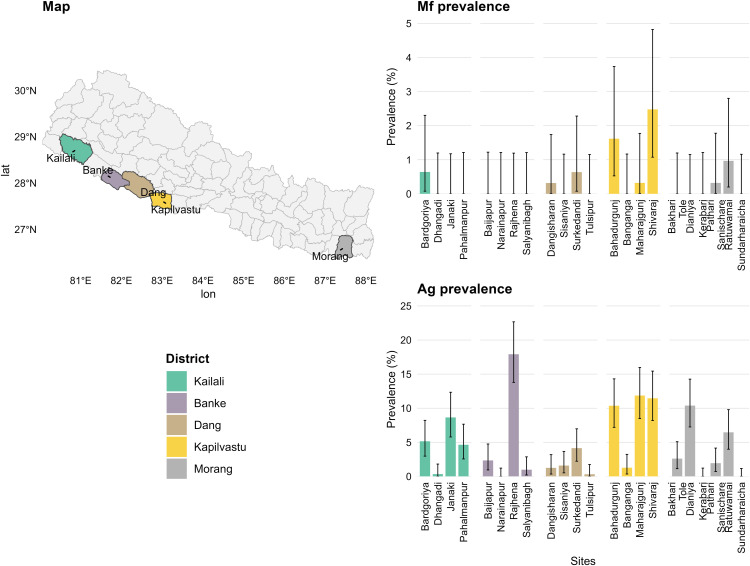
Spatial distribution of lymphatic filariasis (LF) survey districts and site-level antigen (Ag) and microfilaria (Mf) prevalence in Nepal. The map shows the five Nepalese districts included in the LF survey (Kailali, Banke, Dang, Kapilvastu, and Morang), with districts containing LF data highlighted in colour. Site-level LF prevalence is presented for each district representing Mf prevalence (top) or Ag prevalence (bottom). Within each panel, bars show the prevalence (%) recorded at each sampling site, with colours matching the corresponding district on the map. Error bars indicate exact 95% binomial confidence intervals based on the number of positive samples out of the total examined. Base map: Nepal administrative boundaries from the Global Administrative Areas database (GADM v4.1; https://gadm.org/data.html), accessed via the R package geodata. License: https://gadm.org/license.html.

The survey involved 6829 participants aged 20 years and above. The study districts and the site-specific prevalence of Ag and Mf in the 11 EUs are given in Fig 1. All data are shown in S1 Fig and S2 Fig by antigen and microfilariae status respectively.

Site-level coverage of ≥ 65% was evident in the most recent round of IDA MDA across several sites in the EUs (S3 Table). These sites successfully passed the EMS as the microfilariae prevalence was found to be < 1%. Coverage of ≥80% was observed in Dangisharan and Sisania (Dang B), Salyanbagh and Narainapur (Banke A), Baijapur and Rajhena (Banke B), Pahalmanpur (Kaialali A) and Janaki (Kaiali B). Except for Dangisharan (Dang B), all these sites recorded 0% microfilariae positivity. Sites such as Sisaniya (Dang B), Salyanbagh and Narainapur (Banke), Baijapur Rajhena (Banke) and Pahalmanpur (Kailali A), which had ≥ 90% coverage in the recent round of MDA, also recorded 0% microfilariae positivity. Some sites such as Sundarharaicha (Morang A), Patharisanischare (Morang B), Bakharitol (Morang C), and Dhangadhi (Kailali), passed the EMS despite having <65% coverage in the recent round (Table 1). Some sites like Shivraj (Kapilvastu A) and Bahadurgunj (Kapilvastu B) had microfilariae prevalence ≥1% with MDA coverage <65% and thus these sites failed EMS.

**Table 1:** Site-specific antigen (Ag) and microfilariae (Mf) prevalence (%) and mass drug administration (MDA) coverage in the last round with their Epidemiological Monitoring Survey (EMS) result.

SN	Name EU	Name of site	Total sample	Before IDA LF Ag %	# (%) Ag +ve	# (%) Mf +ve	% Mf +ve of Ag +ve	MDA coverage (%)	EMS results
1	Morang-A	Kerabari	302	4.45%	0 (0%)	0 (0%)	0.00	65.89	Pass
		Sundarharaicha	316		0 (0%)	0 (0%)	0.00	43.35	
2	Morang-B	Ratuwamai	310		20 (6.45%)	3 (0.97%)	15.00	69.03	Pass
		Pathari Sanischare	311		6 (1.93%)	1 (0.32%)	16.67	57.23	
3	Morang-C	Dianiya	318		33 (10.38%)	0 (0%)	0.00	66.67	Pass
		Bakhari Tole	306		8 (2.61%)	0 (0%)	0.00	57.19	
4	Kapilvastu-A	Banganga	314	9.16%	4 (1.27%)	0 (0%)	0.00	85.00	Fail
		Shivaraj	323		37 (11.46%)	8 (2.48%)	21.62	47.00	
5	Kapilvastu-B	Bahadurgunj	309		32 (10.36%)	5 (1.62%)	15.63	51.00	Fail
		Maharajgunj	312		37 (11.86%)	1 (0.32%)	2.70	67.95	
6	Dang-A	Surkedandi	314	4.73%	13 (4.14%)	2 (0.64%)	15.38	69.43	Pass
		Tulsipur	318		1 (0.31%)	0 (0%)	0.00	78.93	
7	Dang-B	Dangisharan	318		4 (1.26%)	1 (0.31%)	25.00	86.48	Pass
		Sisaniya	315		5 (1.59%)	0 (0%)	0.00	91.43	
8	Banke –A	Salyanibagh	303	6.84%	3 (0.99%)	0 (0%)	0.00	83.83	Pass
		Narainapur	302		0 (0%)	0 (0%)	N/A	100.00	
9	Banke-B	Baijapur	300		7 (2.33%)	0 (0%)	0.00	94.67	Pass
		Rajhena	307		55 (17.92%)	0 (0%)	0.00	95.11	
10	Kailali-A	Bardgoriya	311	2.51%	16 (5.14%)	2 (0.64%)	12.50	72.12	Pass
		Pahalmanpur	302		14 (4.64%)	0 (0%)	0.00	99.34	
11	Kailali-B	Dhangadi	306		1 (0.33%)	0 (0%)	0.00	59.68	Pass
		Janaki	312		27 (8.65%)	0 (0%)	0.00	87.50	

Overall, 9 out of 11 EUs across the 5 districts passed EMS after two rounds of IDA MDA, indicating infection prevalence below the transmission threshold. Despite LF antigenemia prevalence being ≥2% in 9 sites, microfilariae prevalence remained <1% with IDA MDA coverage ≥65%. Bakharitol, although showing <1% microfilariae and ≥ 2% LF antigenemia, had inadequate coverage of LF MDA. A negative correlation was found between epidemiological coverage in the last round of MDA and microfilariae prevalence (p-value 0.04) in the 22 sites, but no relationship was found between the coverage in the last round of MDA and antigen prevalence (p-value 0.8) (see S3 Fig and S4 Fig). Overall microfilariae positivity was 7.12% among LF antigen-positive individuals and the ratio of antigen-positivity to microfilariae-positivity was 14:1.

There was an overrepresentation of women participants in the sample, with 67.1% being female. The proportion of males was lower across all age groups compared to females (S5 Fig; S5 Table). The median age was 40 years (IQR: 30–55). The overall mean age of the participants was 43, whereas the mean age of individuals who tested positive for LF antigen was significantly higher by approximately 3 years compared to those testing LF antigen negative, with a mean age of 46 (t = -3.2, df = 343, p value = 0.002), and those microfilaria positive were higher still at 49 years (S6 Table), but not significantly different to those testing negative for microfilariae (S6 Fig; t = -0.72, df = 24, p-value = 0.48). These results were consistent with those using Wilcoxon rank sum test with continuity correction. Prevalence by age class (e.g., 20–29, etc.) were not significantly different, but age class prevalence estimates had wide confidence intervals (S6 Fig and S7 Fig; S5 Table). Age was not a significant predictor in binary logistic regression (S8 Table; p-value 0.4).

The survey found females were 50% less likely to report having never been treated compared to males (OR 0.50; 95% CI 0.42-0.59) (S7 Table). At the same time, females were 33% less likely to report non-treatment in the most recent round compared to males (OR 0.67; 95% CI 0.6-0.75) (S8 Table).

**Fig 2:**
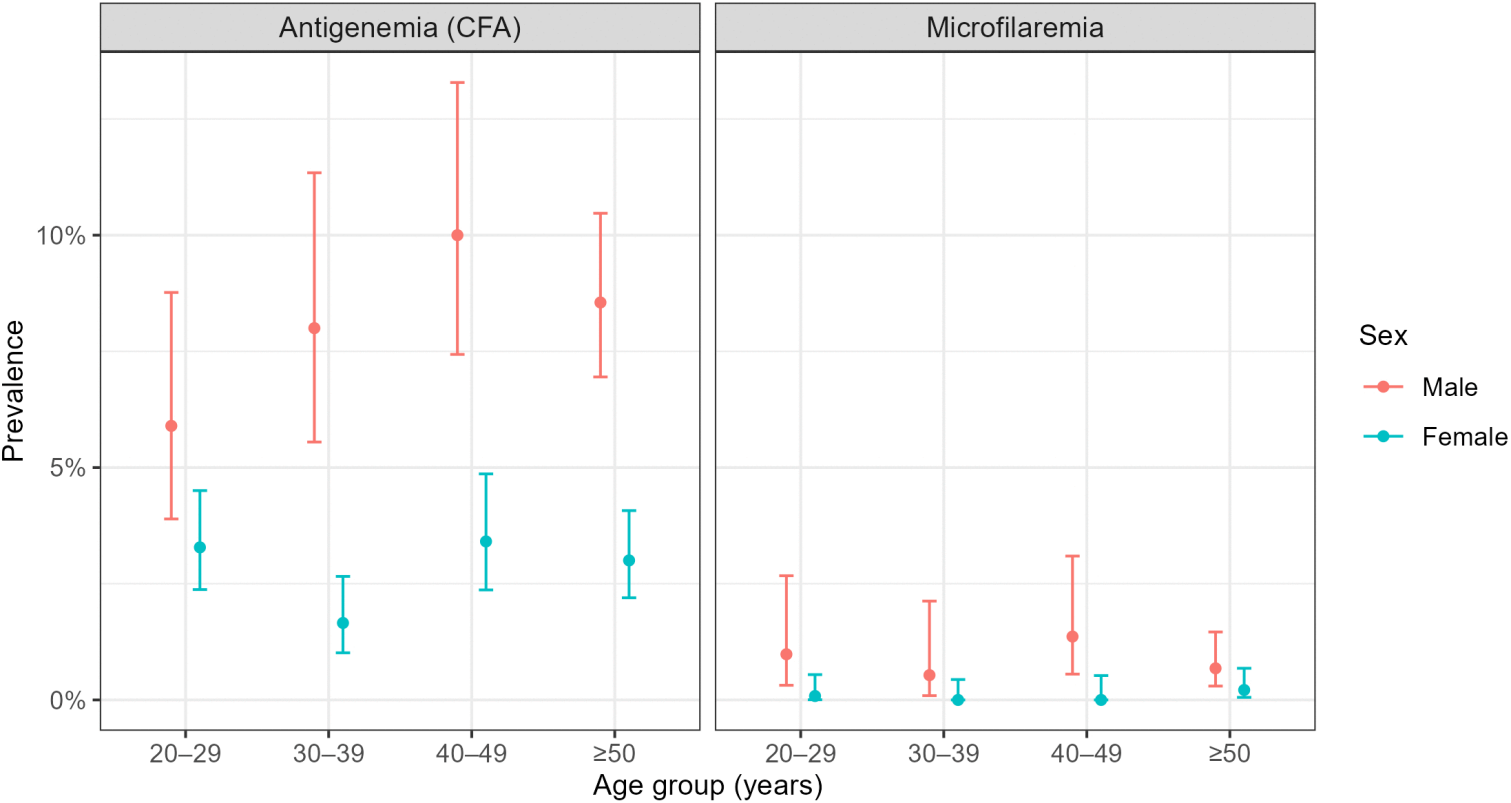
Age- and sex-specific prevalence of LF antigenemia and microfilaremia. Error bars represent 95% Wilson score binomial confidence intervals. Being male was the only significant predictor (β = 1.13, p-value <0.05) in the multi-level glmm with random district effects, but with a p-value close to 0.05 (0.048). Neither age (β = 0.007, p-value 0.6) nor MDA treatment (β = -0.81, p-value 0.08) was significant.

Using univariate analyses, we found that gender (OR 0.12; 95% CI 0.04-0.36) and treatment in the most recent MDA round (OR 0.37; 95% CI 0.15-0.77) were significantly associated with reduced microfilariae rates in the community (S9 Table, Fig 2). Panels show the prevalence of (A) LF antigenemia (circulating filarial antigen, CFA) and (B) microfilaremia among adults aged ≥20 years, stratified by age group and sex. Points represent observed prevalence in each stratum, with vertical lines showing 95% binomial confidence intervals. Prevalence is calculated as the proportion of tested individuals with a positive antigen or microfilaria result in each age–sex group

The Odds ratios (OR) from univariate, individual logistic regressions are shown in Table 2. We observed complete separation for district in the univariate Mf models because several districts had zero or near-zero Mf-positive individuals. This produced unstable and non-interpretable logistic regression estimates (infinite or extremely large ORs), which is expected with sparse binary outcomes. For this reason, and to avoid misleading results, we have omitted district from the univariate Mf OR Table 2.

**Table 2:** Univariate odds ratios for predictors of LF antigenemia and microfilaremia. Odds ratios and 95% confidence intervals (CI) were estimated from univariate logistic regression models for antigenemia (CFA positivity) and microfilaremia. For age groups, the reference category was 20–29 years. For sex, the reference category was male. For MDA participation, the reference group was individuals who did not participate in the most recent MDA round. OR <1 indicates reduced odds of infection relative to the reference category.

Measure	Predictor	OR	95% CI	p-value
**Antigenemia (CFA)**	Age 30–39	0.83	0.56–1.21	0.33
	Age 40–49	1.43	1.02–2.02	0.04
	Age ≥ 50	1.38	1.02–1.88	0.04
	MDA last round taken	0.71	0.56–0.92	0.008
	Sex: Female	0.32	0.26–0.41	<0.001
**Microfilaremia**	Age 30–39	0.50	0.07–2.46	0.43
	Age 40–49	1.01	0.29–3.66	0.99
	Age ≥ 50	0.97	0.33–3.22	0.95
	MDA last round taken	0.35	0.15–0.83	0.016
	Sex: Female	0.28	0.08–0.76	0.023

**Fig 3:**
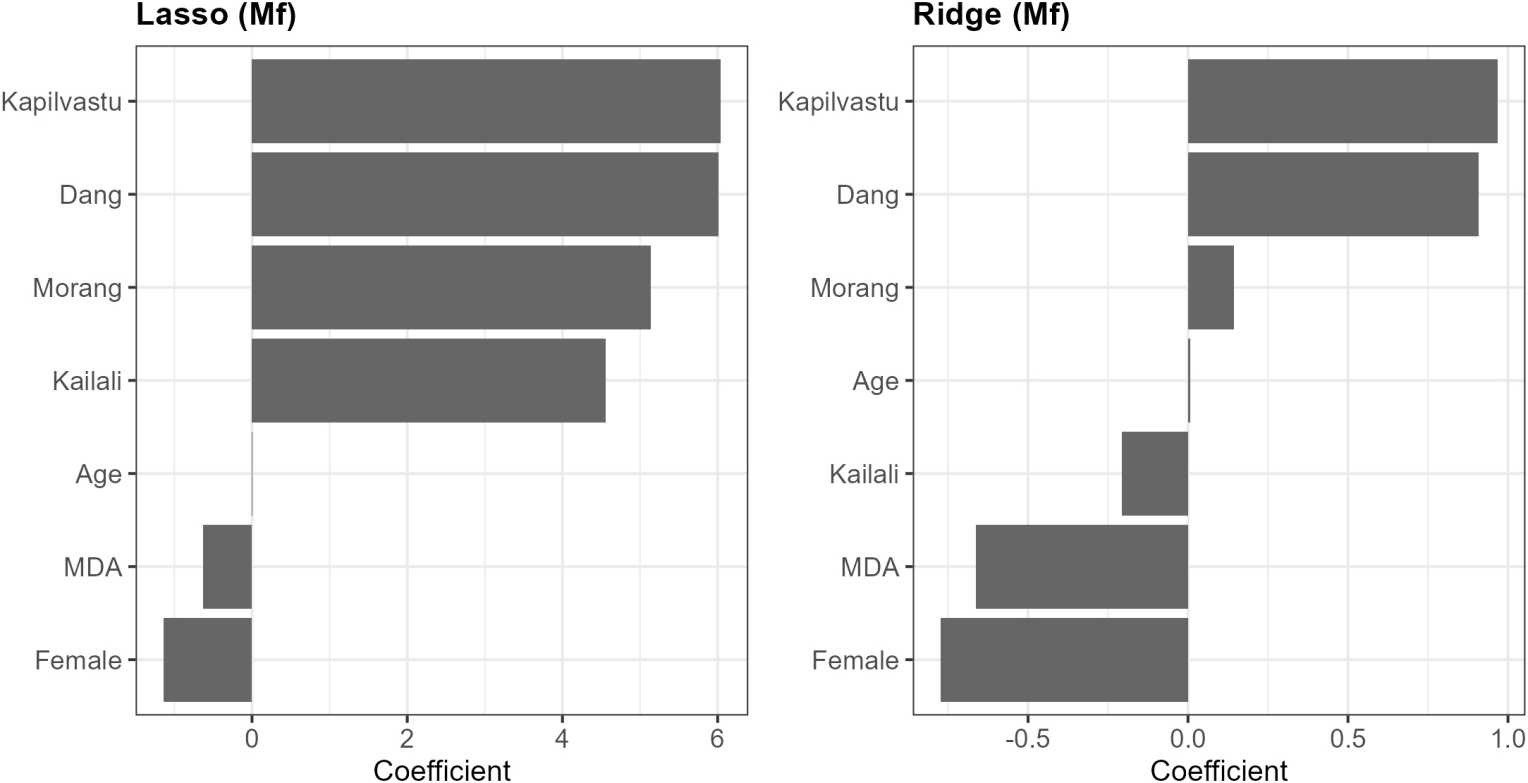
Penalised logistic regression identifying predictors of microfilaremia. Positive coefficients correspond to increased odds of microfilaremia relative to the reference category for each variable, and negative coefficients indicate reduced odds. Confidence intervals are not shown for ridge and lasso because penalisation precludes standard inferential uncertainty estimates.

Multivariate analyses, however, suggested there were multiple interactions as the best models by AIC included all covariates, including districts. Ridge and Lasso regressions showed that being male and living in Kapilvastu were the most important predictors of being *antigen* positive. Being treated in MDA was the most important protective factor, along with relatively lower rates in Morang and Dang districts (S8 Fig). However, for *microfilariae* infection (Fig 3), the most important relative predictors were being from Kapilvastu and Dang and being male (S9 Fig). Those treated in MDA were 66% less likely to be microfilaria positive (OR 0.34, 95% CI 0.15-0.77), and being treated in MDA was the most important protective factor. Age was not important in either analysis accounting for these factors. However, the relative importance of participants being from Kailali switched between analyses (Fig 3).

Ridge and lasso logistic regression models were fitted with microfilaria positivity as the outcome and sex, age, district, and recent MDA participation as predictors. Coefficients are shown on the log-odds scale; larger absolute coefficients indicate stronger associations with microfilaremia.

## Discussion

We conducted a cross-sectional, community-based EMS to assess the prevalence of LF antigenemia and microfilaremia to assess the impact of the IDA regimen in the districts. The survey involved 6,829 participants aged ≥20 years from 11 EUs across five districts. Nine EUs (82%) passed the EMS, as each recorded microfilaria (Mf) prevalence below the WHO threshold and were therefore eligible to proceed with IIS. In contrast, 2 of the 11 EUs (18%) failed the EMS because they recorded an Mf prevalence of ≥1% (the WHO-recommended threshold) in at least one site in each EU. As part of the operational survey recommendations, we suggest conducting two additional rounds of IDA-based MDA with high coverage ensured through directly observed treatment (DOT).

The WHO's New M&E Guidance on Monitoring and Epidemiological Assessment of Mass Drug Administration identifies that the age group ≥ 20 years carries the highest microfilariae burden and represents the greatest risk for propagating LF in the community [12]. The selection of this target age group is also consistent with previous work [19–21]. The guidance also identifies that a lack of microfilariae in adults is a good indicator that there is no ongoing transmission of LF in the community.

Five (23%) out of 22 sites from 11 EUs showed less than 65% LF MDA coverage, a target coverage at implementation unit level for LF elimination. The remaining sites all met the target coverage [9,12], with nine (41%), and five sites (23%) achieving ≥80% and ≥90% MDA coverage respectively. The two EUs which failed EMS had higher LF antigen prevalence in 2022, i.e., before starting 2 rounds of IDA, and less than 65% LF MDA coverage at the site-level in the recent round of MDA. The prevalence of microfilaremia is statistically associated with LF MDA treatment in the recent round, with an odds ratio of 0.34 (95% CI 0.15-0.77), showing the impact of MDA comparable with previous studies [19,22–24]. These results highlight the need for ongoing work to strengthen MDA to eliminate transmission, particularly in sites with higher transmission. A field trial in India with IDA reported 84% clearance of microfilariae [25]. The protective effect of the recent round of IDA-based MDA was lower than that reported in some studies [26–28], but comparable to findings from others, such as the 2019 study in Côte d’Ivoire [29]. Therefore, given the association with IDA and microfilaria positivity, and the Lasso and ridge regression results, the infection persistence in the EUs with evidence of continued transmission despite IDA MDA is likely due to suboptimal coverage (<65%).

Overall microfilariae positivity was 7.12% among LF antigen-positive adults and the ratio of antigen-positive to microfilariae-positivity was found to be 14:1. The lower proportion of Mf positives among Ag positives corroborates earlier studies of IDA [19,30]. A single worm or worms of either sex that do not produce microfilaria [30–32] can remain amicrofilaremic. Microfilaria may be cleared within one month of taking MDA, yet the antigen may persist for years [33] as treatment has limited effect on adult worms. Microfilariae-antigen ratios may reflect the parasite dynamics and the effects of treatment on microfilariae [34]. This likely explains why the microfilariae prevalence was significantly lower among participants in the last round of MDA, whereas the small change in antigen prevalence following treatment was not significant, and this may be due to sterilizing effect of treatment. Because of uncertainty regarding participation in MDA, comparing this ratio pre and post MDA may not be useful for programmatic performance. However, WHO assumes a ratio of 2:1 antigen to microfilariae-positivity for the threshold values. Further studies and meta-analysis of data could help to better understand the effect on this ratio in post-MDA surveillance.

More generally, we found no correlation between age and microfilariae prevalence in contrast to other studies, such as in Egypt and Tanzania [19,20]. If transmission has been ongoing and endemic for a long time, all adults may have had similar cumulative exposure to infected mosquitoes, leading to similar prevalence across age groups, though lower prevalence in older age classes may indicate reduced burden due to effective MDA.

We found a lower microfilariae prevalence in females compared to males (Fig 2; OR 0.12, 95% CI 0.04-0.36), and a similar result was found in Tanzania [19]. Exposure to mosquito bites and different participation rates in MDA may have influenced the gender difference seen in this survey. We found that females had significantly fewer ‘never treated’ issues compared to males and in the recent round treatment issues were significantly lower in females compared to males, matching findings in other countries [19,35]. During the survey, the ratio of males in all age groups was lower compared to females. These findings are comparable to other studies with the 7.5% absentee rate, among which 88% were males, and among the male absentees, more than 76% were between 15 and 34 years of age [36,37]. Together, this suggests greater efforts are needed to find, test and/or treat men, as they are both more likely to be infected and less likely to participate in MDA and surveys.

The present survey clearly showed spatial heterogeneity in MDA treatment which require novel strategies to reduce the treatment gap in subsequent two additional MDA rounds in repeat MDAs in the EMS failed districts. It is also necessary to identify and address other potential barriers to participation, devise the most effective messages and channels for conveying health information, and devise effective drug administration strategies before undertaking the additional MDA rounds [38]. High-risk groups (never treated and elderly) should be identified and targeted, as advocated by Lau et al., 2020, and others [39–41].

Lastly, despite the large sample size, the infection prevalence was low, making it challenging to analyze interactions among predictor covariates and potential confounders, including accounting for spatial clustering. Future analyses could better address multiple comparison issues (i.e., Type I error) and might benefit from the use of other multi-level models (such as hierarchical or mixed-effects models) to analyze additional data. These models would help avoid pseudo-replication and allow for more robust inferences about variation at different levels and interactions among variables. In this study, we used logistic regression without interactions (S9 Table), as well as ridge and Lasso regression (Fig 3), to handle the large but sparse data matrices. However, other approaches, including Bayesian methods, could also be considered.

Repeat surveys in sites that recorded prevalence above the threshold, and surveys in the additional spot-check sites when another site is below the threshold, are recommended in the new WHO national elimination programmes as a response to the outcomes of the EMS [12]. Some elements of bias in this exercise cannot be ruled out as there are possibilities of more efforts to improve coverage in such sites, besides the operational challenges. The results of IIS surveys that continue after EMS to inform stopping decisions may reflect the impact of the IDA-MDA. The results of EMS and IIS1 can be compared to gain a better understanding of the impact of MDA of this clustered disease, once the results are available. It is also recommended that the programme should be prepared for the next round of MDA in the case of unsuccessful EMS, as the preparatory time is short, given that EMS is conducted 9 months after the last IDA-MDA.

Large scale demonstration of the provisional WHO guidelines of monitoring and evaluation in the present study can be considered a strength of the study. It also provided an opportunity to discuss the results in relation to the recent WHO guidelines [12]. This study has some limitations, as it was carried out based on the WHO provisional guideline of LF elimination. This study was carried out before the new WHO guideline and therefore not all the recommendations were performed. Also, two rounds of IDA-MDA were completed only in 2025, and the programme can follow the new recommended actions in response to the results of the previous EMS. It is too early to act in response to the results of current EMS. There were some limitations due to the absences of some Ag positive cases, as Mf surveys were done in the night during separate house visits. It was hard to complete the survey before 10.00 PM, when many people normally retire to bed in the study localities:

This study was also limit because MDA participation was self-reported and assessed approximately nine months after drug administration, which may have introduced recall bias. Molecular xenomonitoring (MX) was also not conducted. MX could have provided additional confirmation of ongoing transmission and strengthened interpretation of the EMS findings; however, it was not feasible due to the absence of a standardized WHO protocol at the time of the study and operational constraints.

## Conclusion

The EMS conducted across 11 evaluation units in 5 Nepali districts revealed that 9 out of these 11 units achieved a microfilariae prevalence of less than 1%, suggesting that the infection prevalence is below the transmission threshold. The low prevalence of microfilariae is attributed to factors such as higher MDA coverage in the campaign and a higher proportion of female participants. LF MDA treatment was notably higher among females compared to males with correspondingly higher microfilariae prevalence among men, suggesting greater efforts may be needed to ensure men are treated in the future. Similarly, some locations have higher transmission and should be targeted. The recent triple-drug regimen demonstrated a significant reduction in LF prevalence, suggesting that current programme strategies are effective and capable of interrupting transmission. However, persistent microfilariae prevalence in two EUs indicates incomplete programme impact, likely related to suboptimal treatment compliance. Two additional rounds of MDA with directly observed treatment are recommended to improve adherence and ensure interruption of transmission.

## Supporting information

S1 Fig All data plotted with the number of *antigen* positive cases in each class. Mass Drug Administration (MDA); Age; Gender; District; and the antigen result (Ag) are shown. These are the hypothesized important predictors (covariates) of LF infection in this study.
(DOCX)

S2 Fig All data plotted with the number of *microfilaria* positive cases in each class. Mass Drug Administration (MDA); Age; Gender; District; and microfilariae (Mf) are shown. These are the hypothesized important predictors (covariates) of LF infection in this study.
(DOCX)

S3 Fig Scatterplot of the epidemiological coverage in the last round of Mass Drug Administration (MDA) (x-axis) as a percentage for each selected sentinel and spot check site and the *microfilaria* (Mf) prevalence as a percentage. A Locally Estimated Scatterplot Smoothing (LOESS) non-parametric regression smooth line is fit through the points with 95% confidence limits shown along with Kendall’s τ (tau) regression results.
(DOCX)

S4 Fig Scatterplot of the epidemiological coverage in the last round of Mass Drug Administration (MDA) (x-axis) as a percentage for each selected sentinel and spot check site and the antigen (Ag) prevalence as a percentage. A Locally Estimated Scatterplot Smoothing (LOESS) non-parametric regression smooth line is fit through the points with 95% confidence limits shown along with Kendall’s τ (tau) regression results.
(DOCX)

S5 Fig Percentage of female participants by age class. (DOCX)

S6 Fig Prevalence of *antigen* positive (A) and *microfilaria* positive (B) cases by age category. Not the different y-axis scales.
(DOCX)

S7 Fig Prevalence of *antigen* positive cases with 95% confidence intervals by age category and district. (DOCX)

S8 Fig Relative importance of being *antigen* positive using Lasso and Ridge regression. See the main text for details.
(DOCX)

S9 Fig Prevalence of *microfilaria* positive cases with 95% confidence intervals by age category and district using those *antigen* positives as the *denominator.* (DOCX)

S1 Table Sentinel and spot check site details. See also S2 Table.
(DOCX)

S2 Table Evaluation unit (EU) summary information with baseline LF *antigen* prevalence. (DOCX)

S3 Table Historical epidemiological coverage data in EUs (Districts). (DOCX)

S4 Table Sentinel and spot-check sites detailed information. (DOCX)

S5 Table Age and gender distribution of participants. (DOCX)

S6 Table Microfilaria infected prevalence by age category. (DOCX)

S7 Table Gender vs MDA compliance. (DOCX)

S8 Table Gender vs MDA compliance in the most recent treatment round. (DOCX)

S9 Table Predictors of microfilariae rate in the community using logistic regression. (DOCX)
