# Supplementary material for: Impact of mass drug administration with Ivermectin, Diethylcarbamazine, and Albendazole in elimination of lymphatic filariasis in five districts of Nepal
Source: PLOS Glob Public Health. 2026 Apr 24;6(4):e0004809. doi: 10.1371/journal.pgph.0004809 (PMC13108797; doi:10.1371/journal.pgph.0004809)
Supplement: S2 Fig — Mass Drug Administration (MDA); Age; Gender; District; and microfilariae (Mf) are shown. These are the hypothesized important predictors (covariates) of LF infection in this study. (DOCX) [file pgph.0004809.s002.docx]

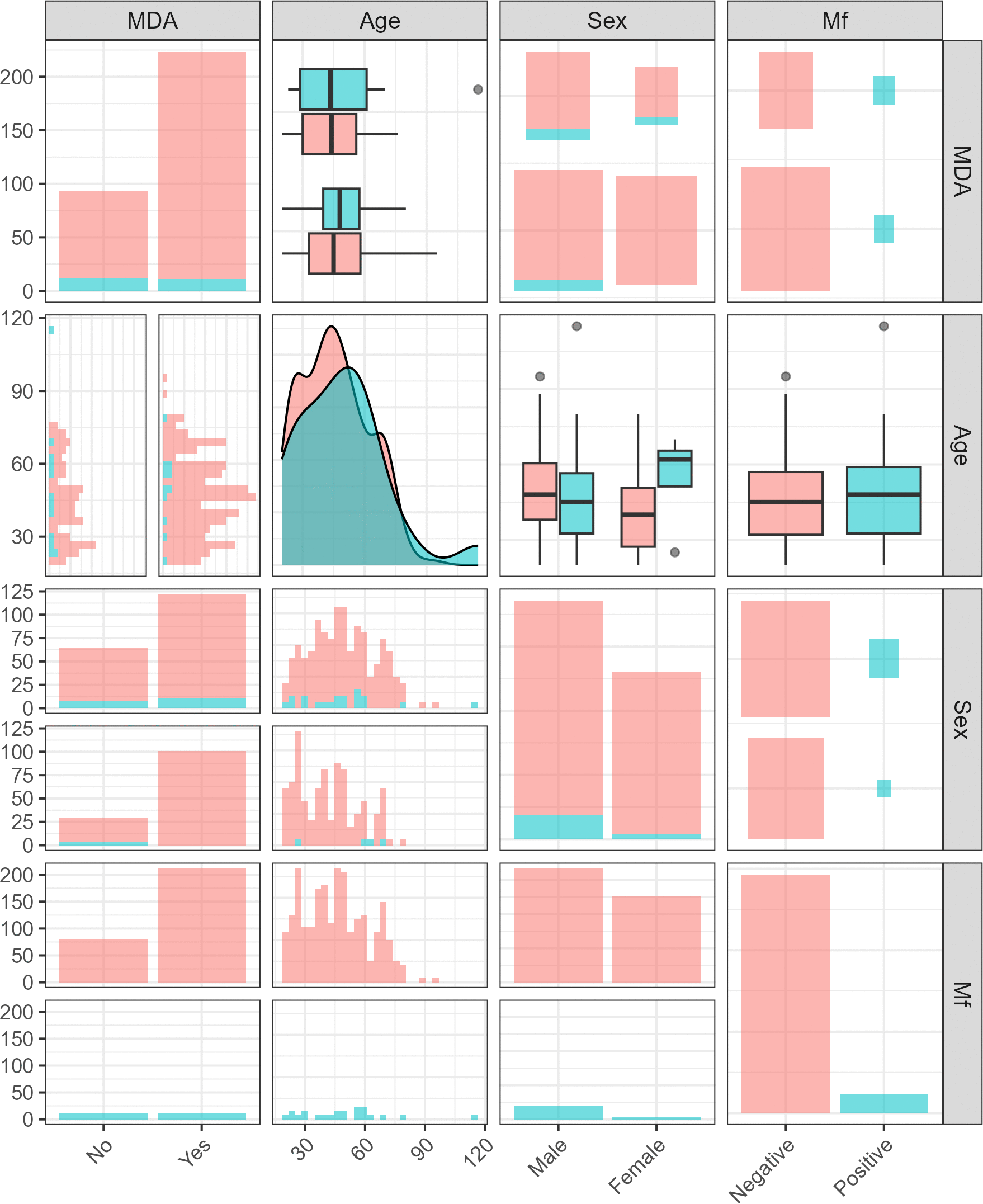


**S2 Fig.** All data plotted with the number of *microfilaria* positive cases in each class. Mass Drug Administration (MDA); Age; Gender; District; and the microfilaria (Mf) are shown. These are the hypothesized important predictors (covariates) of LF infection in this study.
