# Supplementary material for: Impact of mass drug administration with Ivermectin, Diethylcarbamazine, and Albendazole in elimination of lymphatic filariasis in five districts of Nepal
Source: PLOS Glob Public Health. 2026 Apr 24;6(4):e0004809. doi: 10.1371/journal.pgph.0004809 (PMC13108797; doi:10.1371/journal.pgph.0004809)
Supplement: S3 Fig — A Locally Estimated Scatterplot Smoothing (LOESS) non-parametric regression smooth line is fit through the points with 95% confidence limits shown along with Kendall’s τ (tau) regression results. (DOCX) [file pgph.0004809.s003.docx]

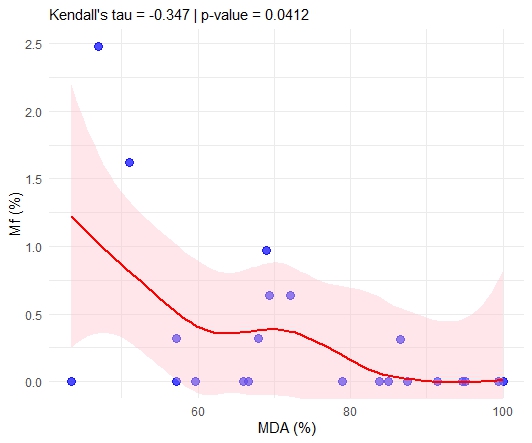


**S3 Fig.** Scatterplot of the epidemiological coverage in the last round of Mass Drug Administration (MDA) (x-axis) as a percentage for each selected sentinel and spot check site and the *microfilaria* (Mf) prevalence as a percentage. A Locally Estimated Scatterplot Smoothing (LOESS) non-parametric regression smooth line is fit through the points with 95% confidence limits shown along with Kendall’s τ (tau) regression results.
