# Supplementary figures and images for: Impact of mass drug administration with Ivermectin, Diethylcarbamazine, and Albendazole in elimination of lymphatic filariasis in five districts of Nepal
Source: PLOS Glob Public Health. 2026 Apr 24;6(4):e0004809. doi: 10.1371/journal.pgph.0004809 (PMC13108797; doi:10.1371/journal.pgph.0004809)

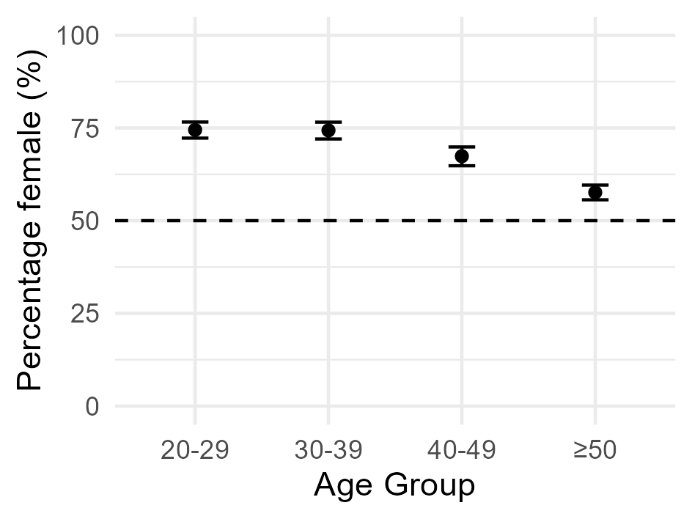


**S5 Fig.** Percentage of female participants by age class

Supplement: S5 Fig — (DOCX) [file pgph.0004809.s005.docx]
