# Supplementary material for: Impact of mass drug administration with Ivermectin, Diethylcarbamazine, and Albendazole in elimination of lymphatic filariasis in five districts of Nepal
Source: PLOS Glob Public Health. 2026 Apr 24;6(4):e0004809. doi: 10.1371/journal.pgph.0004809 (PMC13108797; doi:10.1371/journal.pgph.0004809)
Supplement: S6 Fig — Not the different y-axis scales. (DOCX) [file pgph.0004809.s006.docx]

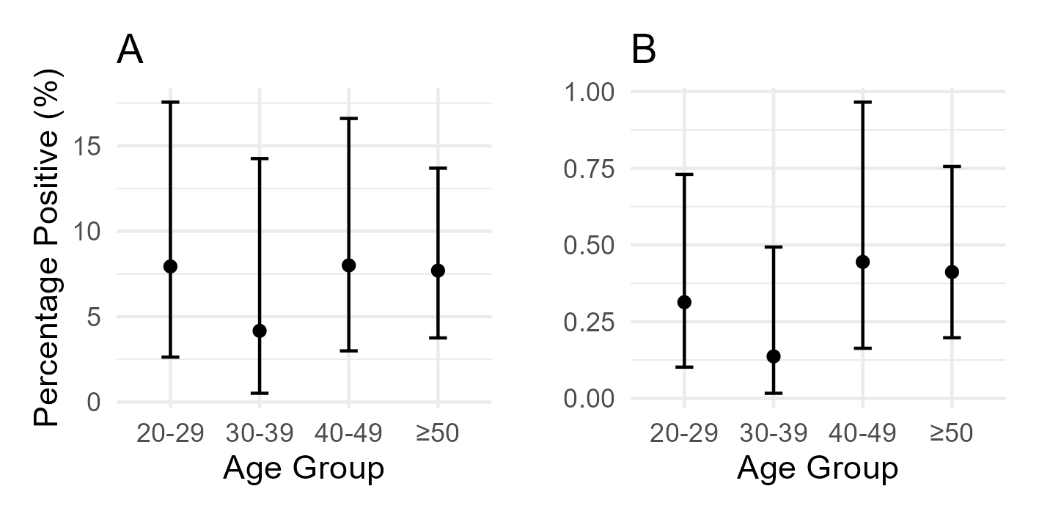


**S6 Fig.** Prevalence of *antigen* positive (A) and *microfilaria* positive (B) cases by age category. Not the different y-axis scales.
