# Supplementary material for: Impact of mass drug administration with Ivermectin, Diethylcarbamazine, and Albendazole in elimination of lymphatic filariasis in five districts of Nepal
Source: PLOS Glob Public Health. 2026 Apr 24;6(4):e0004809. doi: 10.1371/journal.pgph.0004809 (PMC13108797; doi:10.1371/journal.pgph.0004809)
Supplement: S7 Fig — (DOCX) [file pgph.0004809.s007.docx]

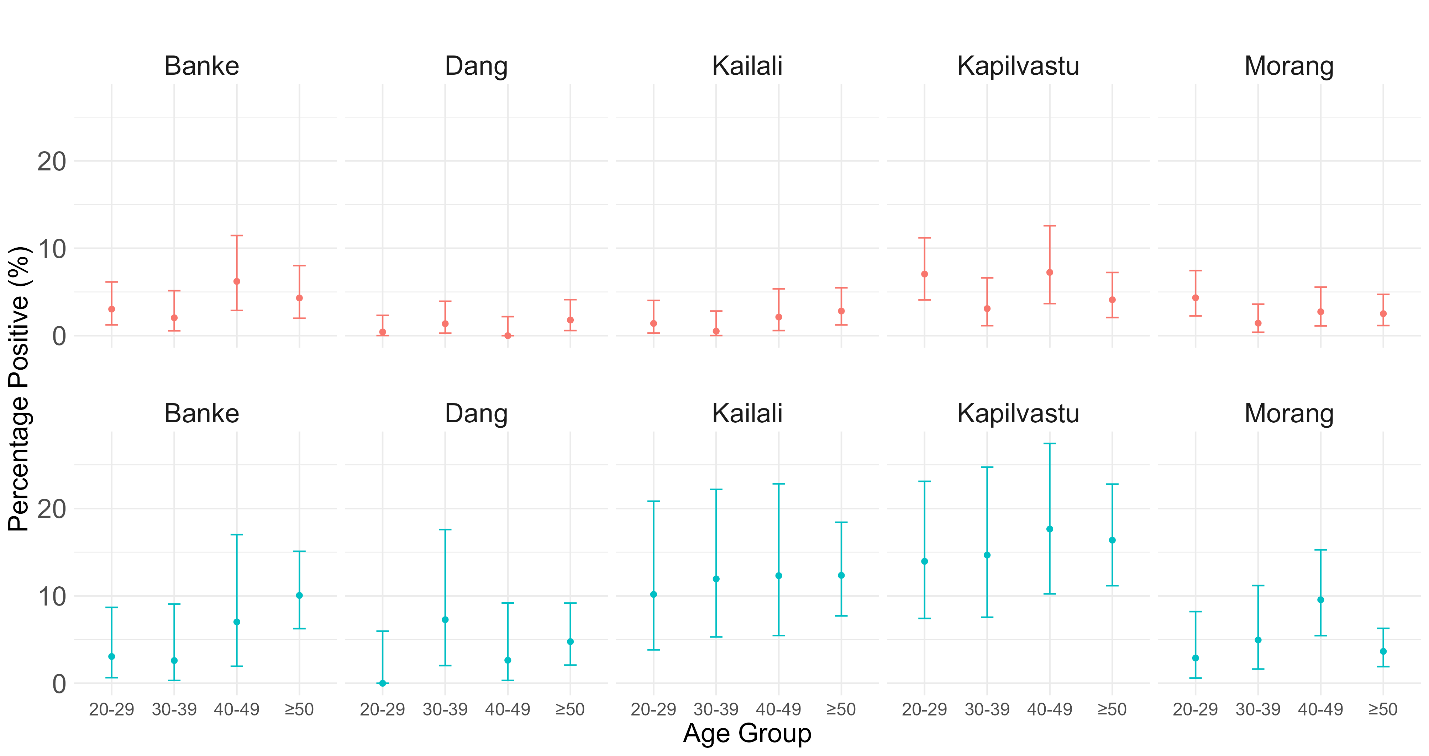


**S7 Fig.** Prevalence of *antigen* positive cases with 95% confidence intervals by age category and district.
