# Supplementary material for: Impact of mass drug administration with Ivermectin, Diethylcarbamazine, and Albendazole in elimination of lymphatic filariasis in five districts of Nepal
Source: PLOS Glob Public Health. 2026 Apr 24;6(4):e0004809. doi: 10.1371/journal.pgph.0004809 (PMC13108797; doi:10.1371/journal.pgph.0004809)
Supplement: S8 Fig — See the main text for details. (DOCX) [file pgph.0004809.s008.docx]

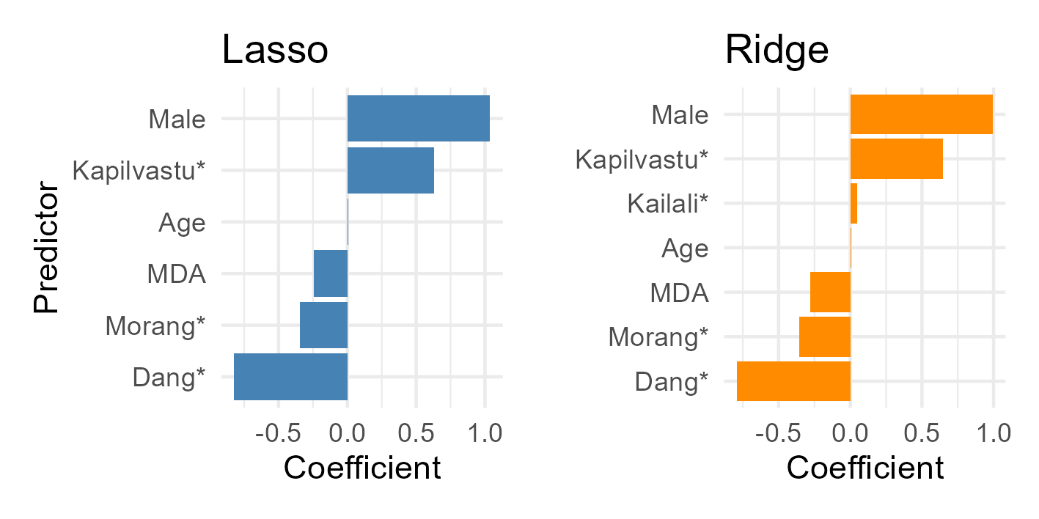


**S8 Fig.** Relative importance of being *antigen* positive using Lasso and Ridge regression. See the main text for details.
