# Supplementary material for: Impact of mass drug administration with Ivermectin, Diethylcarbamazine, and Albendazole in elimination of lymphatic filariasis in five districts of Nepal
Source: PLOS Glob Public Health. 2026 Apr 24;6(4):e0004809. doi: 10.1371/journal.pgph.0004809 (PMC13108797; doi:10.1371/journal.pgph.0004809)
Supplement: S9 Fig — (DOCX) [file pgph.0004809.s009.docx]

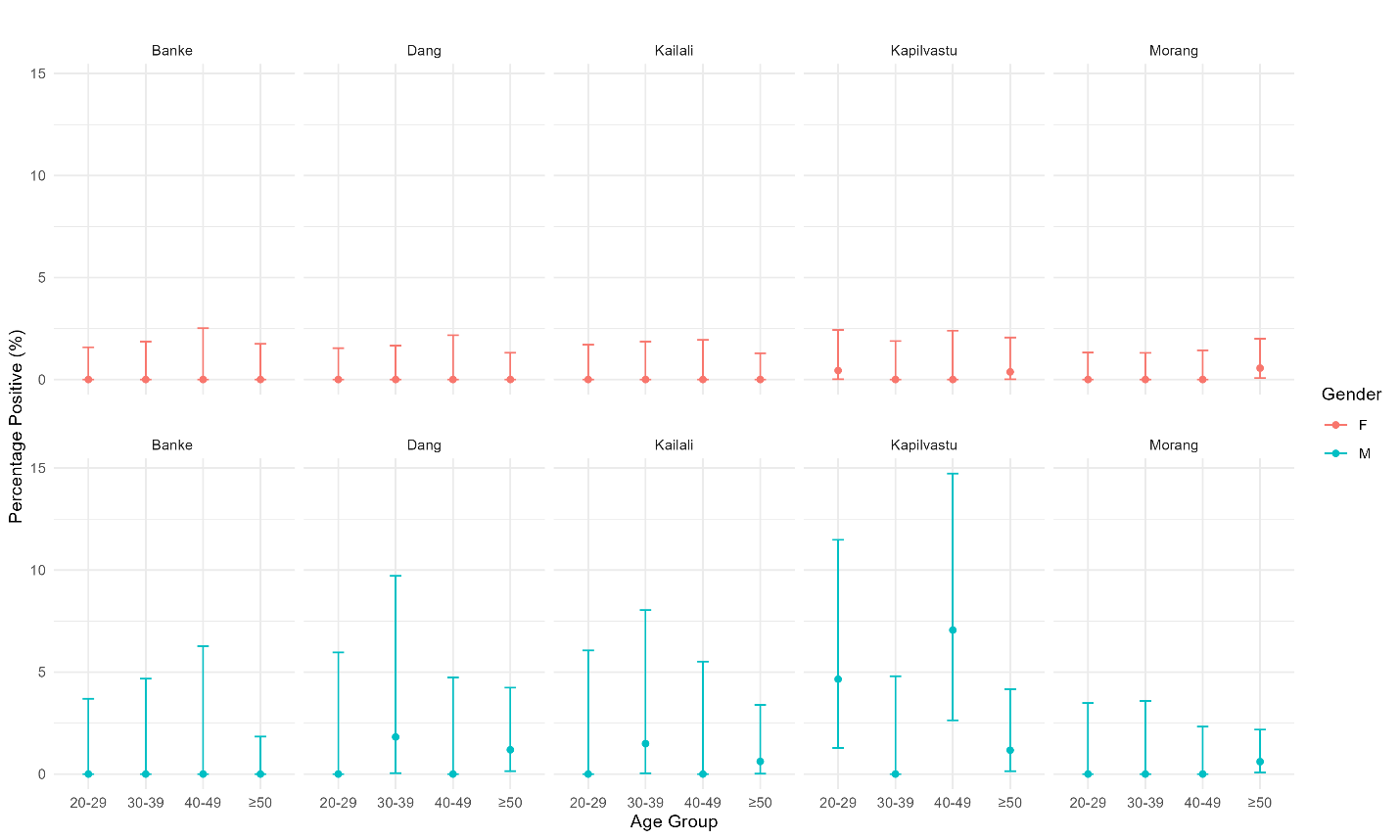


**S9 Fig.** Prevalence of *microfilaria* positive cases with 95% confidence intervals by age category and district using those *antigen* positives as the *denominator*.
