# Supplementary material for: Impact of mass drug administration with Ivermectin, Diethylcarbamazine, and Albendazole in elimination of lymphatic filariasis in five districts of Nepal
Source: PLOS Glob Public Health. 2026 Apr 24;6(4):e0004809. doi: 10.1371/journal.pgph.0004809 (PMC13108797; doi:10.1371/journal.pgph.0004809)
Supplement: S1 Table — See also S2 Table. (DOCX) [file pgph.0004809.s010.docx]

**Supplementary Information**

**S1 Table.** Sentinel and spot check site details. See also S2 Table 2.

| District | Evaluation unit | Sentinel/Spot Check site 1 | Sentinel/Spot Check site 2 |
| --- | --- | --- | --- |
| Morang | Morang-A | Sundarharaicha-5 | Kerabari-6 |
|  | Morang B | Pathari Sanischare-6 | Ratuwamai-7 |
|  | Morang-c | Bakhari Tole-(Biratnagar -12) | Dianiya (Sunawarshi-9) |
| Dang | Dang A | Shivaraj-2 | Banganga-1 |
|  | Dang B | Maharajgunj-1 | Bahadurgunj (Krishnanagar-9) |
| Banke | Banke A | Tulsipur-19 | Surkedandi(Ghorahi-2) |
|  | Banke B | Sisaniya (Rapti-5) | Dangisharan-5 |
| Kapilavastu | Kapilvastu-A | Narainapur -4 | Salyanibagh (Nepalgunj-4) |
|  | Kapilvastu-B | Rajhena (Kohalpur 6) | Baijapur (Raptisonari-4) |
| Kailali | Kailali A | Pahalmanpur (Ghodaghodi-11) | Bardgoriya-2 |
|  | Kailali B | Janaki-1 | Dhangadi-12 |
