# Supplementary material for: Impact of mass drug administration with Ivermectin, Diethylcarbamazine, and Albendazole in elimination of lymphatic filariasis in five districts of Nepal
Source: PLOS Glob Public Health. 2026 Apr 24;6(4):e0004809. doi: 10.1371/journal.pgph.0004809 (PMC13108797; doi:10.1371/journal.pgph.0004809)
Supplement: S2 Table — (DOCX) [file pgph.0004809.s011.docx]

**Supplementary Information**

**S2 Table.** Evaluation unit (EU) summary information with baseline LF *antigen* prevalence.

| **EU/District** | **Municipalities in the EU** | **EU* Baseline %** | **EU Population** | **# Sentinel Sites** | **# Spot Check Sites** |
| --- | --- | --- | --- | --- | --- |
| Morang_A | Miklajung, Letang, Kerabari, Sundarharaincha & Budhiganga | 13.6 | 283996 | 0 | 2 |
| Morang_B | Katahari, Gramthan, Belbari, Kanepokhari, Pathari Sanischare,  Urlabari & Ratuwamai | 13.6 | 422192 | 0 | 2 |
| Morang_C | Sunawarsi, Rangeli, Biratnagar, Dhanpalthan & Jahada | 13.6 | 459704 | 0 | 2 |
| **Morang** | **17 Municipalities (3 EUs)** | **13.6** | **1165892** | **0** | **6** |
| Kapilbastu_A | Banganga, Buddhabhumi, Shivaraj & Bijayanagar | 24 | 309878 | 0 | 2 |
| Kapilbastu_B | Krishnanagar, Maharajgunj,  Kapilvastu,Yesodhara, Mayadevi & Suddhodhan | 24 | 393308 | 1 | 1 |
| **Kapilbastu** | **10 Municipalities (2 EUs)** | **24** | **703186** | **1** | **3** |
| Dang_A | Bangalachuli, Ghorahi &Tulsipur | 29.8 | 413817 | 0 | 2 |
| Dang_B | Shantinagar, Babai, Dangisharan,  Lamahi, Gadhawa, Rajpur & Rapti | 29.8 | 273960 | 1 | 1 |
| **Dang** | **10 Municipalities (2 EUs)** | **29.8** | **687777** | **1** | **3** |
| Banke_A | Nepalgunj, Duduwa &Narainapur | 20.8 | 259547 | 0 | 2 |
| Banke_B | Rapti Sonari, Kohalpur, Baijanath  Khajura & Janaki | 20.8 | 357148 | 1 | 1 |
| **Banke** | **8 Municipalities (2 EUs)** | **20.8** | **616695** | **1** | **3** |
| Kailali_A | Mohanyal, Chure, Godawari, Gauriganga, Ghodaghodi, Bardagoriya &Lamki Chuwa | 6 | 431306 | 1 | 1 |
| Kailali_B | Janaki, Joshipur, Tikapur, Bhajani,  Kailari & Dhangadhi | 6 | 495582 | 0 | 2 |
| **Kailali** | **13 Municipalities (2 EUs)** | **6** | **926888** | **1** | **3** |
| **5 districts** | **58 Municipalities (11 EUs)** |  |  | **4** | **18** |

*****District baseline information is reflected as EU baseline information.
