# Supplementary material for: Impact of mass drug administration with Ivermectin, Diethylcarbamazine, and Albendazole in elimination of lymphatic filariasis in five districts of Nepal
Source: PLOS Glob Public Health. 2026 Apr 24;6(4):e0004809. doi: 10.1371/journal.pgph.0004809 (PMC13108797; doi:10.1371/journal.pgph.0004809)
Supplement: S3 Table — (DOCX) [file pgph.0004809.s012.docx]

**Supplementary Information**

**S3 Table**. Historical epidemiological coverage data in EUs (Districts)

| **Districts/EUs** |  | | **MDA coverage in (%)** | | | | | | | | | | | | | | |
| --- | --- | --- | --- | --- | --- | --- | --- | --- | --- | --- | --- | --- | --- | --- | --- | --- | --- |
|  | **Year of MDA Rounds** | | | | | | | | | | | | | | | | |
|  | **2007** | **2009** | | **2010** | **2011** | **2012** | **2013** | **2014** | **2015** | **2016** | **2017** | **2018** | **2019** | **2020** | **2021** | **2022-IDA** | **2023-IDA** |
| **Morang_A, B & C** |  |  | |  |  | 70.4 | 71.3 | ***58.1*** | ***63.3*** | 70.8 | 72.3 | ***59.5*** | ***64.5*** | 68.5 | 73.6 | **80.1** | **80.8** |
| **Kapilbastu_A & B** | 81.2 | 81.9 | | 85.9 | 82.9 | 83.9 | 87.1 | 81.0 | 79.1 | No MDA | 79.3 | 71.6 | ***56.6*** | 83.7 | 87.2 | **84.1** | **80.1** |
| **Dang_A & B** |  |  | | 87.4 | ***58.3*** | ***65.0*** | 68.6 | ***54.5*** | ***57.1*** | 72.7 | 72.2 | ***56.2*** | 65.3 | 67.5 | 68.6 | **82.3** | **87.4** |
| **Banke_A & B** |  |  | | 85.7 | ***47.4*** | ***63.0*** | 67.1 | 71.0 | 65.4 | 67.1 | 65.9 | ***55.0*** | 67.1 | 70.4 | 67.9 | **72.4** | **85.6** |
| **Kailali_ A & B** |  |  | |  |  |  | 66.6 | 68.5 | 69.3 | 69.5 | 69.1 | 69.6 | 69.9 | 73.2 | 76.0 | **79.6** | **82.3** |

*Morang, Kapilbastu, Dang, Banke, and Kailali have completed two rounds of IDA MDA after failing pre-TAS in 2021. District Coverage data used for EU coverage information.
