# Supplementary material for: Impact of mass drug administration with Ivermectin, Diethylcarbamazine, and Albendazole in elimination of lymphatic filariasis in five districts of Nepal
Source: PLOS Glob Public Health. 2026 Apr 24;6(4):e0004809. doi: 10.1371/journal.pgph.0004809 (PMC13108797; doi:10.1371/journal.pgph.0004809)
Supplement: S4 Table — (DOCX) [file pgph.0004809.s013.docx]

**Supplementary Information**

**S4 Table.** Sentinel and spot-check sites detailed information

| **SN** | **Districts** | **Evaluation Units** | **Name of Sentinel/ Spot Check Site (Municipalities/wards** | **Sentinel/ Spot check site** | **Population of the site** | **Reason for site selection** |
| --- | --- | --- | --- | --- | --- | --- |
| 1 | Morang | **1. Morang_A** | Sundarharaicha-5 (101) | New spot check site | 6255 | Low MDA coverage and a high number of cases reported during morbidity mapping |
|  |  |  | Kerabaari-6 (102) | New spot check site | 3416 | Low MDA coverage and a high number of cases reported during morbidity mapping |
|  |  | **2. Morang_B** | Pathari Sanischare-6 (201) | New spot check site | 6062 | Low MDA coverage (75%) which is the lowest in this ward of Municipality and high number of cases (mostly hydrocele) reported during morbidity mapping. |
|  |  |  | Ratuwamai - 7 (202) | New spot check site | 6508 | Reported cases are high during morbidity mapping. |
|  |  | **3. Morang_C** | Biratnagar-12 (Bakhri tole) (301) | New spot check site | 19006 | Low MDA coverage |
|  |  |  | Dainiya, Sunwarshi-9 (302) | Spot Check | 8129 | Old, failed SC site (7.8% Ag in 2021) |
| 2 | Kapilbastu | **4. Kapilbastu_A** | Banganga, Banganga Municipality-1 (401) | New spot check site | 8721 | Reported cases are high during morbidity mapping. |
|  |  |  | Shivraj -2 (402) | New spot check site | 5680 | Reported cases are high during morbidity mapping. |
|  |  | **5. Kapilbastu_B** | Maharajgunj-1 (501) | Sentinel | 7322 | Old, failed S site (7.9% antigen +ve in 2021) |
|  |  |  | Bahadurgunj, Krishnanagar-9 (502) | Spot check | 6644 | Old, failed SC site (9.1% antigen +ve in 2021) |
| 3 | Dang | **6.Dang_A** | Tulsipur-19 (601) | New spot check site | 8107 | Low MDA coverage |
|  |  |  | Surkedandi, Ghorahi-2 (602) | Spot check site | 8158 | Old, failed spot check site (3.8% antigen +ve in 2021) |
|  |  | **7.Dang_B** | Sisaniya, Rapti-5 (701) | Sentinel | 6386 | Old, failed sentinel site (4.8% antigen +ve in 2021) |
|  |  |  | Dangisharan-5 (702) | Spot Check | 3781 | Reported cases are high during morbidity mapping. |
| 4 | Banke | **8. Banke_A** | Narainapur-4 (801) | New spot check | 6715 | Low MDA coverage and cases high in morbidity mapping. |
|  |  |  | Salyanibagh, Nepalgunj 4 (802) | Spot check | 13526 | Old, failed SC site (2.8% antigen +ve in 2021) |
|  |  | **9. Banke_B** | Rajhena, Kohalpur -6 (901) | Sentinel | 8542 | Old, failed sentinel site (5.8% antigen +ve in 2021) |
|  |  |  | Baijapur, Raptisonari RM-4 (902) | New Spot check | 7847 | Low MDA coverage and cases high in morbidity mapping |
| 5 | Kailali | **10. Kailali_A** | Pahalmanpur, ghodaghodi-11 (1001) | Sentinel | 5085 | Old, failed sentinel site (2.6% antigen +ve in 2021) |
|  |  |  | Bardagoriya-2 (1002) | New Spot check | 8046 | Cases are high in morbidity mapping and adjoining areas of previous spot-check sites (Chuwa). |
|  |  | **11. Kailali_B** | Janaki -1 (1101) | New Spot check | 5406 | Low MDA coverage and cases high in morbidity mapping |
|  |  |  | Dhangadhi -12 (1102) | New Spot check | 12691 | Low MDA coverage and high cases in morbidity mapping |
