# Supplementary material for: Impact of mass drug administration with Ivermectin, Diethylcarbamazine, and Albendazole in elimination of lymphatic filariasis in five districts of Nepal
Source: PLOS Glob Public Health. 2026 Apr 24;6(4):e0004809. doi: 10.1371/journal.pgph.0004809 (PMC13108797; doi:10.1371/journal.pgph.0004809)
Supplement: S5 Table — (DOCX) [file pgph.0004809.s014.docx]

**Supplementary Information**

**S5. Table**. Age and gender distribution of participants.

| **Sex** | **Age group** |  |  |  |  |  |  |  |
| --- | --- | --- | --- | --- | --- | --- | --- | --- |
|  | **20-29** | **30-39** | **40-49** | **≥50** | **Mean (%)** | **Percent females (%)** | **Mean age antigen-positive (95% CI)** | **Mean age Mf positive (95% CI)** |
| **Male** | 406 (25.5%) | 375  (24.9%) | 439 (32.6%) | 1029 (42.4%) | 43.2 | 67.1 | 46.1  (44.2-47.9) | 49.1  (39.8-58.5) |
| **Female** | 1185 (74.4%) | 1087 (75.1%) | 909 (67.4%) | 1399 (57.6%) |  |  |  |  |
| **Total** | 1591 | 1462 | 1348 | 2428 |  |  |  |  |
