# Supplementary material for: Impact of mass drug administration with Ivermectin, Diethylcarbamazine, and Albendazole in elimination of lymphatic filariasis in five districts of Nepal
Source: PLOS Glob Public Health. 2026 Apr 24;6(4):e0004809. doi: 10.1371/journal.pgph.0004809 (PMC13108797; doi:10.1371/journal.pgph.0004809)
Supplement: S6 Table — (DOCX) [file pgph.0004809.s015.docx]

**Supplementary Information**

**S6 Table.** Microfilaria infected prevalence by age category.

| **Age group** | **Positives/total tested** | **Prevalence (95% CI)** |
| --- | --- | --- |
| 20-29 | 5/1595 | 0.31 (0.10-0.73) |
| 30-39 | 2/1462 | 0.14 (0.02-0.49) |
| 40-49 | 6/1349 | 0.45 (0.16-0.97) |
| ≥50 | 10/2428 | 0.41 (0.20-0.76) |
