# Supplementary material for: Impact of mass drug administration with Ivermectin, Diethylcarbamazine, and Albendazole in elimination of lymphatic filariasis in five districts of Nepal
Source: PLOS Glob Public Health. 2026 Apr 24;6(4):e0004809. doi: 10.1371/journal.pgph.0004809 (PMC13108797; doi:10.1371/journal.pgph.0004809)
Supplement: S7 Table — (DOCX) [file pgph.0004809.s016.docx]

**Supplementary Information**

**S7 Table.** Gender vs MDA compliance.

| **Sex** | **Never treated** | **Treated with at least one round** | **Chi-square (χ^2^) p-value** | **Odds ratio (95% CI)** |
| --- | --- | --- | --- | --- |
| **Female** | 305 (6.7%) | 4278 (93.3%) | <0.05 | 0.50 (95% CI, 0.42-0.59) |
| **Male** | 283 (12.6%) | 1968 (87.4%) |  |  |
