# Supplementary material for: Impact of mass drug administration with Ivermectin, Diethylcarbamazine, and Albendazole in elimination of lymphatic filariasis in five districts of Nepal
Source: PLOS Glob Public Health. 2026 Apr 24;6(4):e0004809. doi: 10.1371/journal.pgph.0004809 (PMC13108797; doi:10.1371/journal.pgph.0004809)
Supplement: S8 Table — (DOCX) [file pgph.0004809.s017.docx]

**S8 Table.** Gender vs MDA compliance in the most recent treatment round.

| **Sex** | **No** | **Yes** | **Chi-square (χ^2^) p-value** | **Odds ratio (95% CI)** |
| --- | --- | --- | --- | --- |
| **Female** | 953 (20.8%) | 3630 (79.2%) | <0.05 | 0.67 (95% CI 0.6-0.75) |
| **Male** | 634 (28.2%) | 1617 (71.8) |  |  |
