# Supplementary material for: Impact of mass drug administration with Ivermectin, Diethylcarbamazine, and Albendazole in elimination of lymphatic filariasis in five districts of Nepal
Source: PLOS Glob Public Health. 2026 Apr 24;6(4):e0004809. doi: 10.1371/journal.pgph.0004809 (PMC13108797; doi:10.1371/journal.pgph.0004809)
Supplement: S9 Table — (DOCX) [file pgph.0004809.s018.docx]

**S9 Table.** Predictors of microfilariae rate in the community using logistic regression.

| **Predictor Variables** | **p-value** | **Odds ratio (95% CI)** |
| --- | --- | --- |
| **Age** | 0.421 | 1.010 (95% CI 0.99-1.04) |
| **Gender (female)** | <0.001 | 0.120 (95% CI 0.04-0.36) |
| **Compliance in the recent round** | 0.010 | 0.336 (95% CI 0.15-0.77) |
